# Effect of serum triglyceride level on the prognosis of patients with hepatocellular carcinoma in the absence of cirrhosis

**DOI:** 10.1186/s12944-018-0898-y

**Published:** 2018-11-06

**Authors:** Xiaoli Liu, Mengge Li, Xinhui Wang, Zhibo Dang, Yuyong Jiang, Xianbo Wang, Zhiyun Yang

**Affiliations:** Center for Integrative Medicine, Beijing Ditan Hospital, Capital Medical University, No. 8 Jing Shun East Street, Beijing, 100015 People’s Republic of China

**Keywords:** TG, HCC, Lipid, Lipoprotein, Prognosis

## Abstract

**Background:**

The liver plays an important role in the metabolism of lipid and lipoprotein. Dyslipidemia has been demonstrated to be related with several cancers, but the association between serum lipid and hepatocellular carcinoma (HCC) in the absence of cirrhosis remains unclear.

**Methods:**

A total of 2528 patients with HCC at the Beijing Ditan Hospital between February 2008 and December 2017 were retrospectively included in the study. We identified 200 patients with HCC without cirrhosis by histopathology, imaging, endoscopic findings, and laboratory tests. Multivariate regression analysis was performed to determine the independent characteristics associated with HCC without cirrhosis and its prognosis.

**Results:**

In the logistics regression analysis, compared to patients with HCC with cirrhosis, patients with HCC without cirrhosis were more likely to have elevated triglyceride (TG) levels (OR = 2.66; 95% CI, 1.18–6.01; *P* = 0.019). The Kaplan-Meier analysis revealed that a lower TG level was a risk factor regardless of the presence of cirrhosis. The results of the Cox proportional hazard regression analysis showed that a decreased TG level was significantly related to a worse overall survival (HR = 0.51; 95% CI, 0.29–0.89; *P* = 0.017).

**Conclusion:**

Serum TG level may be an independent factor to predict the prognosis of patients with HCC in the absence of cirrhosis.

**Electronic supplementary material:**

The online version of this article (10.1186/s12944-018-0898-y) contains supplementary material, which is available to authorized users.

## Introduction

Hepatocellular carcinoma (HCC) is the fifth most common malignancy and has the second highest cancer mortality worldwide.[1, 2]. Although most HCCs are usually accompanied by cirrhosis owing to chronic viral infections, a certain number of patients with HCC, ranging widely from 7 to 54%, do not have cirrhosis[3–6]. It is perceived that the liver is one of the most important organs in multiple metabolite pathways[7], including lipid and lipoprotein. Hepatic cellular necrosis caused by cirrhosis and HCC leads to aberrations in serum lipid and lipoprotein levels[8, 9]. Some studies reported that the triglyceride (TG) level significantly decreased in patients with cirrhosis or HCC[10–12]. However, the alterations in the lipid and lipoprotein levels in patients with HCC without cirrhosis remain unclear.

Recently, abnormal lipid and lipoprotein levels were considered to be related with the incidence and development of several types of cancer[13, 14]. Several researches showed that elevated TG level and suppressed high-density lipoprotein cholesterol (HDLc) level were related to a high risk of occurrence and death in colon, breast, lung, and prostate cancers[15–18]. However, the relationships between blood profile and liver cancer were contradictory due to the complex etiology. Several studies that considered the risk factor of non-alcoholic fatty liver disease (NAFLD) revealed that elevated TG and low HDLc levels attribute to a greater risk of HCC in patients with cirrhosis[19]. In contrast, regarding HBV and HCV infection, TG levels were inverse contributory factors for HCC[20, 21]. Furthermore, the prognostic effect of lipid profiles on HCC remains unclear. Few studies indicated that low cholesterol and HDLc levels could predict the recurrence of HCC in patients after liver resections[22]. However, whether the alterations of lipid profiles are correlated with HCC prognosis is unclear.

In this study, we compared the clinical and laboratory characteristics of patients with HCC with and without cirrhosis and found that high TG levels were independently related to HCC without cirrhosis. Furthermore, by multiple analyses, we identified high TG level to be an independent prognostic factor for better survival.

## Materials and methods

### Patients

**Fig. 1:**
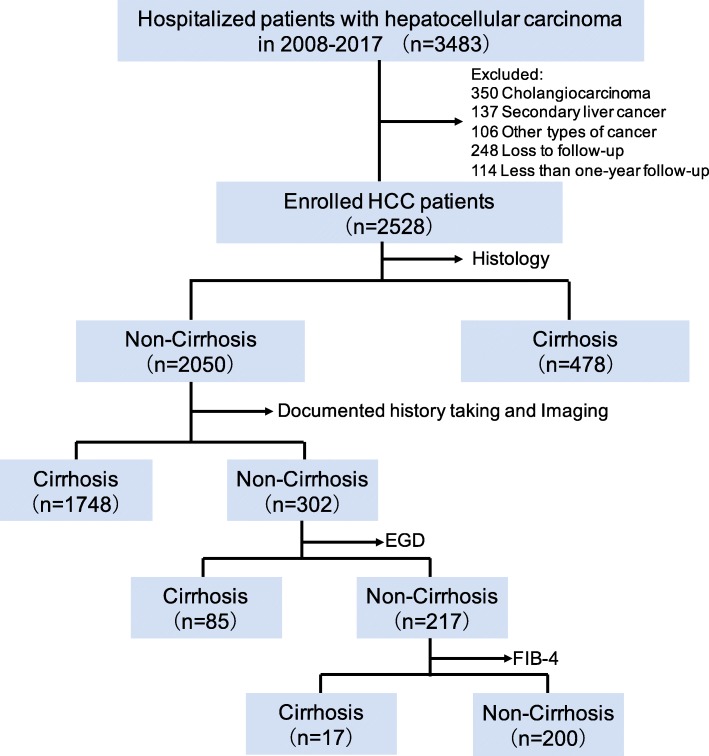
Outline of the classification of HCC cohort study. Determination of cirrhosis status followed by laboratory tests, pathology, endoscopy and imaging studies. EGD, Esophagogastroduodenoscopy

A total of 3483 patients diagnosed with HCC in the medical records from February 2008 to December 2017 at the Beijing Ditan Hospital, Capital Medical University (Beijing, China) were retrospectively enrolled in the study. The study was approved by the Committee of Ethics at Beijing Ditan Hospital, Capital Medical University. The inclusion criteria were as follows: (1) HCC diagnosed based on the pathology or serum α-fetoprotein (AFP) level of ≥400 ng/mL in combination with imaging that showed typical appearances of HCC; (2) confirmed tumor staging of HCC, based on the Barcelona-Clinic Liver Cancer (BCLC) staging system; (3) had complete clinical data; (4) more than 1 year of follow-up. We excluded 955 patients with cholangiocarcinoma, metastatic liver cancer, or other types of cancer; those lost to follow-up, and those with short follow-up period (Fig. 1). We divided the final 2528 patients into subjects with and without cirrhosis according to the results of histopathology, clinical presentation, radiological studies, endoscopic examinations, and laboratory tests at the time of HCC diagnosis. We classified patients without cirrhosis if they meet the following criteria: 1) had liver biopsy-documented non-cirrhosis histology within 1 year before HCC diagnosis; 2) did not have clinical complications consisting of ascites, variceal bleeding, hepatorenal syndrome, and hepatic encephalopathy; 3) did not have cirrhosis related to morphologic changes and portal hypertension on imaging examination; 4) did not have esophageal and gastric varices on upper endoscopy; and 5) had FIB-4 value of < 1.45. We calculated the FIB-4 score using laboratory results within 1 year before HCC diagnosis using the following formula: age (years) × aspartate aminotransferase (U/L)/platelet (PLT) (10^9^/L) × alanine aminotransferase (U/L)^1/2^.

### Statistical analysis

The statistical analyses were performed using SPSS version 21.0 software. Data were expressed as median and range for non-normal distribution and mean ± standard deviation (SD) for normal distribution. Comparisons of patient characteristics were analyzed using Student’s t-test for normal distribution and Mann-Whitney U test for non-normal distribution. Categorical variables were assessed by Pearson X^2^ or Fisher’s exact test. One-way ANOVA test followed by Tukey’s multiple comparison test was performed for lipid profiles across the 3 groups (no cirrhosis, compensated cirrhosis, and decompensated cirrhosis). For all analyses, *P* values of < 0.05 were considered statistically significant.

The univariate and multivariate logistic regression analyses were performed to identify factors that were independently associated with patients with HCC without cirrhosis. We used Kaplan-Meier curves to estimate overall survival (OS) and progression-free survival (PFS) of different groups and compared survival curves using the log-rank test. The Cox proportional hazard regression analysis was used to determine the variables associated with the prognosis in patients with HCC without cirrhosis.

## Results

### Baseline characteristics

Between 2008 and 2017, we identified 2528 patients with HCC, of whom 2328 (92.1%) had cirrhosis before or at the time of HCC diagnosis and 200 (7.9%) did not have cirrhosis. Figure 1 shows the flowchart used to classify patients into the cirrhosis categories.

The demographic characteristics and clinical and biochemical characteristics of patients with and without cirrhosis are presented in Table 1. At the time of HCC diagnosis, patients without cirrhosis were 3.6 years younger than those with cirrhosis. Patients without cirrhosis had two times greater proportions of family history of HCC and history of hyperlipidemia than those without cirrhosis. Regarding the etiology, a greater proportion of patients with HCC without cirrhosis had NAFLD and idiopathic condition with no etiologic risk factor, and a lower proportion had alcoholic liver disease compared with patients with cirrhosis. In all patients with HCC, HBV and HCV infections were both great risk factors. Patients with HCC in the absence of cirrhosis had a larger proportion of solitary tumor nodule (72% vs 55.1%, *p* < 0.0001) and a significantly lower prevalence of portal vein thrombosis (PVTT) at the time of HCC diagnosis (7.0% vs 20.6%, *p* < 0.0001) compared to those with underlying cirrhosis. The diameter of tumor showed no difference between HCC patients without cirrhosis (median, 3.5 cm) and patients with cirrhosis (median, 3.1 cm) (*p* = 0.213). Patients with HCC in the absence of cirrhosis were more likely to have BCLC stage 0-A tumor (vs C or D) when compared with patients with cirrhosis. Moreover, HCC patients without cirrhosis received more resection treatment and less palliative care than patients with cirrhosis (*p* = 0.002). The median MELD scores were lower in patients with HCC without cirrhosis (median score, 3.4) than those with cirrhosis (median score, 5.39) (*p* < 0.0001). There were high levels of leukocyte counts, platelet counts, albumin, triglyceride, and prothrombin activity and low ratio of neutrophil to lymphocyte and low levels of aspartate aminotransferase (AST), total bilirubin (TBIL), and γ-glutamyl transferase (GGT) in patients with HCC without cirrhosis (*p* < 0.0001). There were no differences in other patient characteristics by cirrhosis status.

**Table 1:** Demographic data and clinical characteristics of patients with hepatocellular carcinoma

	Non-cirrhosis *n* = 200 (%)	Cirrhosis *n* = 2328 (%)	*P* values
Age(mean ± SD)	53.36 ± 11.94	56.96 ± 10.34	< 0.0001
Gender (male)	164 (82.0)	1793 (77.0)	0.106
Family history of HCC			0.005
Yes	14 (7.0)	74 (3.2)	
No	186 (93.0)	2254 (96.8)	
Smoking			0.131
Smoker	91 (45.5)	932 (40.0)	
Non-smoker	109 (54.5)	1396 (60.0)	
Alcohol			0.759
alcohol	79 (39.5)	894 (38.4)	
No alcohol	121 (60.5)	1434 (61.6)	
Diabetes			< 0.0001
Yes	21 (10.5)	506 (21.7)	
No	179 (89.5)	1822 (78.3)	
Hypertension			0.81
Yes	50 (25)	600 (25.8)	
No	150 (75)	1728 (74.2)	
Hyperlipidemia			0.002
Yes	25 (12.5)	154 (6.6)	
No	175 (87.5)	2174 (93.4)	
Coronary artery disease			0.918
Yes	5 (2.5)	61 (2.6)	
No	195 (97.5)	2267 (97.4)	
Etiology			0.003
HBV	153 (76.5)	1823 (78.3)	
HCV	23 (11.5)	177 (7.6)	
Alcohol abuse	10 (5.0)	248 (10.7)	
NAFLD	4 (2.0)	25 (1.1)	
idopathic	10 (5.0)	55 (2.4)	
HBeAg at baseline			0.005
Negative	98 (54.1)	1289 (60.6)	
Positive	45 (24.9)	572 (26.9)	
Missing data	38 (21.0)	265 (12.5)	
HBV-DNA at baseline			0.024
Low(< 500 IU/ml)	71 (41.3)	791 (39.8)	
High(> 500 IU/ml)	63 (36.6)	894 (45.0)	
Missing data	38 (22.1)	301 (15.2)	
Antiviral therapy			0.972
Yes	146 (79.3)	1686 (79.0)	
No	29 (15.8)	348 (16.3)	
Missing data	9 (4.9)	99 (4.6)	
Tumor diameter (cm)	3.5 (2.3, 6.2)	3.1 (2.0, 5.7)	0.213
Tumor multiplicity			< 0.0001
solitary	136 (72)	1259 (55.1)	
multiple	53 (28)	1024 (44.9)	
PVTT at baseline			< 0.0001
Yes	14 (7.0)	479 (20.6)	
No	186 (93.0)	1849 (79.4)	
AFP (ng/ml)			0.754
AFP < 400	148 (74.0)	1746 (74.9)	
AFP ≥ 400	52 (26.0)	634 (25.1)	
BCLC staging			< 0.0001
0-A	106 (53.0)	835 (37.3)	
B	73 (36.5)	734 (31.9)	
C	18 (9.0)	433 (17.8)	
D	3 (1.5)	326 (13.0)	
Treatment for HCC			0.002
Resection	30 (15.0)	185 (7.9)	
Minimally invasive	132 (66.0)	1578 (67.8)	
Palliative	38 (19.0)	565 (24.3)	
MELD scores	3.4 (1.29,5.30)	5.39 (2.36,8.48)	< 0.0001
Leukocyte counts (10^9^/L)	4.43 (5.71,7.25)	3.05 (4.32,5.90)	< 0.0001
NLR	2.19 (1.55,3.68)	2.44 (1.62,3.97)	0.019
Platelets (10^9^/L)	151.8 (112.5197.08)	87.25 (57.23,136.5)	< 0.0001
ALT (U/L)	32.1 (22.72,56.7)	33.2 (22.3,54.2)	0.9
AST (U/L)	31.15 (23.8,54.03)	42.4 (28.83,72.13)	< 0.0001
Totall Bilirubin (umol/L)	13.4 (9.7,16.98)	19.85 (13.2,32.48)	< 0.0001
γ-GGT (U/L)	44.9 (25.9,94.33)	59.35 (33.22,123.8)	< 0.0001
Albumin (g/L)	40.09 ± 4.80	35.014 ± 6.34	< 0.0001
Triglyceride (mmol/L)	1.009 ± 0.49	0.87 ± 0.45	< 0.0001
Prothrombin activity (%)	88.77 ± 14.51	75.06 ± 18.00	< 0.0001
Child Staging			< 0.0001
A	177 (88.5)	1125 (48.3)	
B	20 (10.0)	877 (37.7)	
C	3 (1.5)	326 (14.0)	

Abbreviations: SD, standard deviation; PVTT, portal vein tumor thrombus; AFP, alpha-fetoprotein; NLR, Neutrophil-lymphocyte ratio; ALT, alanine aminotransferase; AST, aspartate aminotransferase; γ-GGT γ-glutamyl transferase

### Factors associated with HCC without cirrhosis

Table 2 shows the results of the univariate and multivariate logistic regression analyses. Compared to patients with HCC with cirrhosis, patients with HCC without cirrhosis were statistically significantly more likely to be younger (≤50 years), in Child A stage and have a family history of HCC and PVTT in the baseline. Patients with HCC without cirrhosis also frequently had higher platelet counts (≥100*10^9^/L), higher TG level (≥1.71 mmol/L), and more normal GGT level (< 60 U/L) (*P* < 0.05 for all comparisons). Furthermore, we compared the lipid parameters in HCC patients with different status of cirrhosis and found the TG decreased in decompensated cirrhosis than non-cirrhosis and compensated cirrhosis (P < 0.05, Additional file 1: Table S1).

**Table 2:** Factors associated with hepatocellular carcinoma in the absence of cirrhosis

Variables	Univariate analysis			Multivariate analysis		
	OR	95%CI	*P* values	OR	95%CI	*P values*
Age > 50 yrs	0.46	0.34–0.62	< 0.0001	0.42	0.25–0.70	< 0.0001
Gender (male)	1.36	0.94–1.98	0.11			
Family history of HCC	2.29	1.27–4.14	0.006	2.91	1.07–7.92	0.037
Diabetes	0.59	0.39–0.89	0.012			
Hyperlipidemia	2.02	1.29–3.16	0.002			
Etiology						
HBV	0.79	0.54–1.13	0.19			
HCV	1.43	0.92–2.22	0.12			
Alcohol abuse	0.44	0.23–0.85	0.014			
NAFLD	6.77	2.0–23.32	0.002			
HBV-DNA ≥ 500 IU/ml	0.79	0.55–1.11	0.18			
Antiviral therapy	1.04	0.69–1.57	0.86			
PVTT at baseline	0.29	0.17–0.51	< 0.0001			
Leukocyte counts ≥4*10^9^/L	2.31	1.79–2.98	< 0.0001			
PLT ≥100*10^9^/L	6.23	4.31–9.02	< 0.0001	6.67	3.32–13.40	< 0.0001
AST ≥ 40 (U/L)	0.52	0.39–0.70	< 0.0001			
γ-GGT ≥ 60 (U/L)	0.57	0.42–0.77	< 0.0001	0.46	0.27–0.78	0.004
TG ≥ 1.71 mmol/L	2.46	1.50–4.02	< 0.0001	2.66	1.18–6.01	0.019
PTA < 70%	0.18	0.11–0.29	< 0.0001			
AFP ≥ 400 ng/ml	1.05	0.76–1.47	0.75			
Child staging			< 0.0001			
A	17.1	5.43–53.88	< 0.0001	5.67	1.34–24.02	0.019
B	2.48	0.73–8.40	0.15			
C (Reference)						
BCLC staging			< 0.0001			
0-A	13.8	4.35–43.77	< 0.0001			
B	10.81	3.38–34.54	< 0.0001			
C	4.52	1.32–15.47	0.016			
D (Reference)						

Abbreviations: *PTA* Prothrombin activity

### Survival analysis

**Fig. 2:**
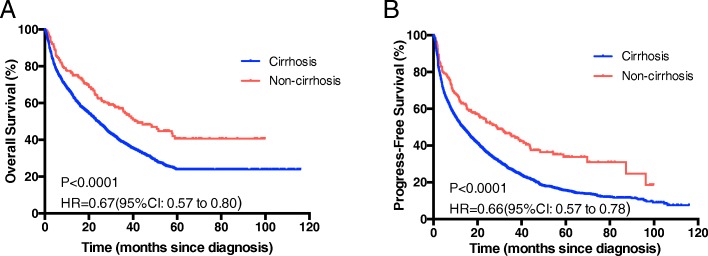
Overall survival (**a**) and Progress-free survival (**b**) in HCC patients with and without cirrhosis

**Fig. 3:**
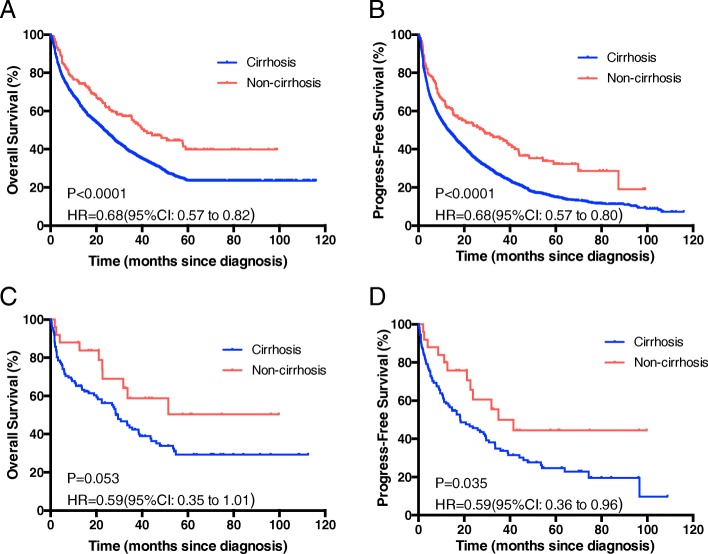
Overall survival (OS) and Progress-free survival (PFS) in different triglyceride subgroups of HCC patients with and without cirrhosis. **a-b** The OS (**a**) and PFS (**b**) in triglyceride level < 1.7 mmol/L subgroup. **c-d** The OS (**c**) and PFS (**d**) in triglyceride level ≥ 1.7 mmol/L subgroup

The median overall survival time was 42 months for non-cirrhosis HCC patients and 25 months for HCC with cirrhosis cohort, respectively. There was a significantly longer OS in patients without cirrhosis compared to patients with cirrhosis (hazard ratio [HR], 0.67; 95% CI, 0.57–0.80; *P* < 0.0001; Fig. 2a); the 1-, 3-, and 5- year OS was 74.46, 55.23 and 40.61%, respectively, in the non-cirrhosis group and 65.22, 38.30, and 23.93%, respectively, in the cirrhosis group. The result of progress-free survival rate (PFS) was consistent with the OS (hazard ratio [HR], 0.66; 95% CI, 0.57–0.78; *P* < 0.0001; Figure 2b). In exploratory subgroup analysis, the group without cirrhosis had an PFS benefit than those with cirrhosis in both subgroups of patients with TG level ≥ 1.71 mmol/L and < 1.71 mmol/L (Fig. 3b and d). Similarly, patients without cirrhosis had significantly better OS than those without cirrhosis in patients with TG < 1.71 mmol/L (Fig. 3a), but the benefit of OS was not remarkable in TG ≥1.71 mmol/L group (*P* = 0.053, Fig. 3c).

### Risk factors of death in patients with HCC without cirrhosis

Table 3 displays the results of univariate and multivariate Cox proportional hazard regression analysis. By univariate analysis, alcohol abuse, presence of PVTT at the time of HCC diagnosis, high TBIL level, high GGT level, AFP level of ≥400 ng/mL, tumor size of ≥5 cm, and tumor number of ≥2 were found to be significant risk factors, and a high albumin level and antiviral therapy were the protective factors for the incidence of death in patients without cirrhosis. By the multivariate analysis, we found that PVTT at the time of HCC diagnosis (adjusted HR, 3.40; 95% CI, 1.73–6.67), GGT (HR, 1.006; 95% CI, 1.004–1.009), and tumor size of > 5 cm (HR, 2.79; 95% CI, 1.72–4.50) remained the independent risk factors associated with a high risk of mortality. Furthermore, antiviral therapy (HR, 0.53; 95% CI, 0.32–0.89) and high serum TG levels (HR, 0.51; 95% CI, 0.29–0.89) were the independent protective factors for the survival of patients with HCC without cirrhosis.

**Table 3:** Factors associated with overall survival of patients with HCC in the absence of cirrhosis

Variables	Univariate analysis			Multivariate analysis		
	HR	95%CI	*P values*	HR	95%CI	*P values*
Age > 50 yrs	1.01	0.99–1.03	0.283			
Gender (male)	0.85	0.51–1.40	0.517			
Family history of HCC	1.16	0.51–2.67	0.72			
Alcohol abuse	1.76	1.18–2.63	0.006			
Diabetes	1.41	0.83–2.38	0.202			
Hypertension	0.85	0.53–1.36	0.507			
Hyperlipidemia	0.98	0.54–1.80	0.949			
Etiology						
HBV	0.62	0.39–0.97	0.037			
HCV	1.8	1.08–3.02	0.025			
Alcohol abuse	1.54	0.71–3.32	0.272			
NAFLD	2.07	0.65–6.54	0.216			
HBV-DNA ≥ 500 IU/ml	1.36	0.88–2.10	0.164			
Antiviral therapy	0.52	0.32–0.86	0.011	0.53	0.32–0.89	0.015
PVTT at baseline	6.17	3.32–11.46	< 0.0001	3.4	1.73–6.67	< 0.0001
Leukocyte counts	1.05	0.96–1.14	0.332			
NLR	0.99	0.95–1.03	0.675			
Platelets (10^9^/L)	1.002	0.999–1.005	0.193			
AST (U/L)	1.002	0.998–1.006	0.403			
Totall Bilirubin (umol/L)	1.005	1.002–1.008	0.001			
Albumin (g/L)	0.939	0.902–0.978	0.002			
γ-GGT (U/L)	1.006	1.004–1.008	< 0.0001	1.006	1.004–1.009	< 0.0001
Triglyceride (mmol/L)	0.7	0.44–1.11	0.128	0.51	0.29–0.89	0.017
PTA (%)	0.986	0.973–0.999	0.032			
AFP ≥ 400 ng/ml	2.235	1.469–3.40	< 0.0001			
Tumor size > 5 cm	3.99	2.65–6.02	< 0.0001	2.79	1.72–4.50	< 0.0001
Tumor numbers ≥2	2.3	1.53–3.46	< 0.0001			
CRP (mg/L)	1.008	1.003–1.013	0.002			

Abbreviations: *CRP* C-reactive protein

**Fig. 4:**
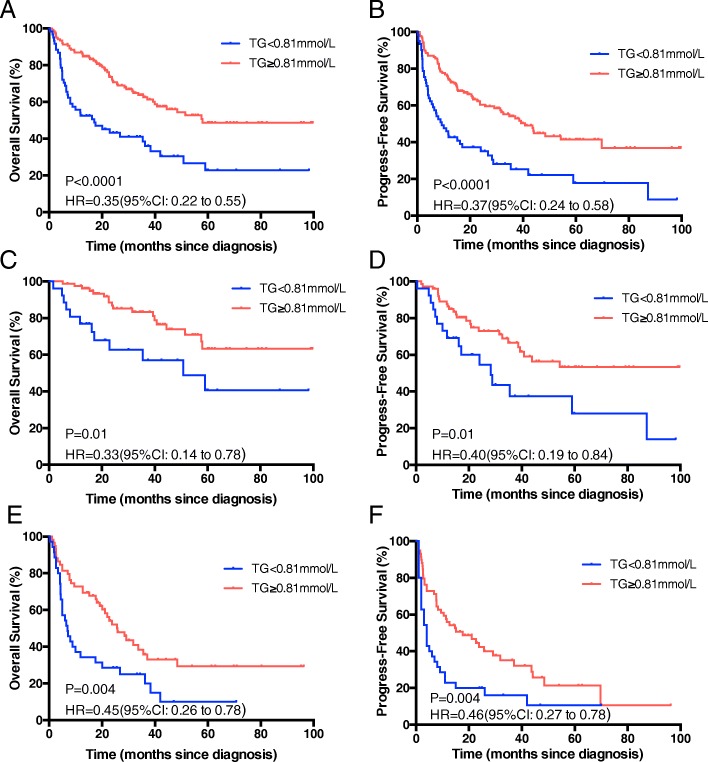
Kaplan-Meier curve analysis showing overall survival (OS) and progress-free survival (PFS) of different triglyceride level in HCC patients without cirrhosis. **a-b** The OS (**a**) and PFS (**b**) for all HCC patients without cirrhosis. **c-d** The OS (**c**) and PFS (**d**) for HCC patients without cirrhosis with BCLC stage 0 and A. **e-f** The OS (**e**) and PFS (**f**) for HCC patients without cirrhosis with BCLC stage B, C and D

A further analysis was performed to evaluate the influence of each risk factor on the OS and PFS of patients with HCC without cirrhosis. We set 0.81 mmol/L as the triglyceride cut-off value which was the largest one for the Youden index. In the Kaplan-Meier analysis presented in Fig. 4, patients with HCC without cirrhosis with TG level < 0.81 mmol/L had worse OS and PFS compared to those with TG level of ≥0.81 mmol/L (*P* < 0.0001; Fig. 4a–b). We also examined the predictive value of serum TG level at different BCLC stages (Fig. 4c–f). For patients in stages 0-A and patients in stages B, C and D, the OS of patients with TG level of < 0.81 mmol/L were significantly lower than those with TG level of ≥0.81 mmol/L throughout the follow-up period (Fig. 4c–f).

Combining the triglycerides level, cirrhosis status and age which are related to the serum triglycerides, it was possible to delineate 8 risk categories, with a 5.80-fold (95% CI 3.19–10.52) increased probability of death in patients with HCC when the highest category (HCC patients with cirrhosis aged above 50 years and with triglycerides < 0.81 mmol/L) was compared with the lowest category (HCC patients without cirrhosis aged < 50 years and with triglycerides ≥0.81 mmol/L) (Table 4).

**Table 4:** Risk of overall survival according to cirrhosis status, age, triglyceride level in patients with hepatocellular carcinoma

	Non-cirrhosis		Cirrhosis	
	Age < 50 yrs	Age ≥ 50 yrs	Age < 50 yrs	Age ≥ 50 yrs
TG < 0.81 mmol/L	7.48 (3.58–15.61) *P* < 0.0001	3.87 (1.87–8.03) *P* < 0.0001	5.65 (3.07–10.39) P < 0.0001	5.80 (3.19–10.52) *P* < 0.0001
TG ≥ 0.81 mmol/L	1^*^	2.93 (1.51–5.68) *P* = 0.001	3.55 (1.93–6.54) *P* < 0.0001	3.76 (2.07–6.82) *P* < 0.0001

Results are expressed as hazard ratios (95% confidence intervals). ^*^ Reference category

## Discussion

Previous studies suggested that dyslipidemia was a newly identified risk factor in the survival of several cancers such as colorectal, breast, and prostate cancers [15, 16, 18]. Although one study demonstrated that preoperative HDL level was a predictor of HCC recurrence after liver resections [22]; recently, there were few studies that addressed the association between lipid profiles and the outcome of liver cancer. In our study, we found that a decreased TG level in the baseline was an independent risk factor of OS in patients with HCC without cirrhosis. A reduced serum TG level was related to worse OS and PFS by the optimal cutoff values using the ROC analysis.

Hypertriglyceridemia was previously considered to be closely related to a higher risk of cardiovascular disease [23]. However, some epidemiological researches have addressed the interrelation between serum TG and cancer risk lately. A high TG level has been linked to increased esophageal and colon cancer risks in a large-scale European cohort [24]. Other studies revealed a high TG level was inversely associated with prostate and breast cancer [25, 26]. It is inconsistent with the relationship between serum TG concentration and HCC risk. A large prospective cohort study with a long follow-up period showed that elevated TG level contributed to an increased risk of primary liver cancer in patients who had more than 50% history of alcohol liver disease [19]. Another study indicated that the TG level was strongly associated with reduced risk of HBV-related HCC [27]. The association between TG and HCC remains unclear. To our knowledge, this is the first time to report a negative association between TG levels and HCC death. In this study, we have shown that patients with HCC in the absence of cirrhosis with TG level < 0.81 mmol/L had a worse OS and PFS than patients with TG level ≥ 0.81 mmol/L (*P* < 0.0001). The normal cutoff values for TG may be interpreted by the fact that the serum TG level decreased by 20–30% in patients with HCC compared to healthy participants [28, 29]. In contrast, the pro-inflammatory cytokines like interleukin-6 (IL-6), IL-1, and tumor necrosis factor alpha (TNF-α) secreted by tumor cells may inhibit TG synthesis [30, 31]. On the contrary, increased adipose TG lipase and hormone-sensitive lipase activity in cancer may promote the complete hydrolysis of TG molecule to free fatty acids (FFA), which can provide substrates for the proliferation of tumor cells [32, 33]. Suppressed synthesis and excessive hydrolysis play an important role in dyslipidemia. Decreased levels of TG have been connected with increased circulating levels of IL-6, TNF-α which are thought to induced the tumor cell proliferation and inhibit apoptosis [34]. In addition, the high death rate linked with decreased TG concentration may be a consequence of cancer-associated cachexia. However, the exact mechanisms require further investigation.

We also found that there were some differences in lipid, lipoproteins, and apolipoproteins in patients with HCC with and without cirrhosis. Patients with HCC without cirrhosis had increased triglyceride, cholesterol, HDL-C, LDL-C, and ApoB levels compared to patients with cirrhosis. More profoundly, the decrease of lipid components was more obvious in patients with HCC with decompensated cirrhosis than those with compensated cirrhosis. These results were consistent with a previous study that lowering of lipoprotein levels was significantly linked to the increasing severity of liver disease [35, 36]. Besides, using multiple logistic regression analysis, portal vein thrombosis and tumor size > 5 cm at HCC diagnosis were associated with poor prognosis, consistent with previous studies in several cancers [37]. We also found that patients with HCC without cirrhosis had elevated serum TG levels (OR = 2.66). The liver plays a key role in the synthesis and metabolism of lipids and lipoprotein. Hepatic FA derive form endogenous lipogenesis and the FFA plasma pool are processed to triacylglycerols and stored or rapidly metabolized in hepatocyte [38]. Liver cirrhosis due to long-term virus infections causes greater hepatocyte necrosis and influences the lipid concentration. Our finding may provide new evidence that patients with cirrhosis developed more serious liver injury and dyslipidemia. The better liver function reflected by a higher TG level may explain the better survival in patients with HCC without cirrhosis than those with cirrhosis.

Our study had some limitations. First, the variables analyzed in our study did not have a treatment strategy, which was an important factor affecting the prognosis. The influence of different clinical interventions on the lipid profiles need to be clarified in future studies. Second, HBV infection is the main risk factor for the incidence of HCC in China. The racial differences in TG levels should be considered, and the cutoff value needs to be redefined when generalizing the results to people with different risk factors for HCC. Finally, the patients in our study were mostly men, and the prognostic effect of TG in women may be limited.

In conclusion, this study revealed that TG levels at the time of HCC diagnosis may be considered as independent prognostic factor for liver cancer. These results indicate that some appropriate treatments may be applied to adjust lipids to normal or high levels.

## Additional file

Additional file 1: **Table S1.** Changes of lipid parameters in HCC patients without cirrhosis, compensated cirrhosis and decompensated cirrhosis (DOCX 14 kb)
