# Supplementary material for: Effect of serum triglyceride level on the prognosis of patients with hepatocellular carcinoma in the absence of cirrhosis
Source: Lipids Health Dis. 2018 Nov 6;17:248. doi: 10.1186/s12944-018-0898-y (PMC6220457; doi:10.1186/s12944-018-0898-y)
Supplement: Supplementary file 1 — Table S1. Changes of lipid parameters in HCC patients without cirrhosis, compensated cirrhosis and decompensated cirrhosis (DOCX 14 kb) [file 12944_2018_898_MOESM1_ESM.docx]

Table S1. Changes of lipid parameters in HCC patients without cirrhosis, compensated cirrhosis and decompensated cirrhosis

| Lipid parameters | Non-cirrhosis n=200 | Compensated cirrhosis n=310 | Decompensated cirrhosis n=2018 | *P* values |
| --- | --- | --- | --- | --- |
| Cholesterol (mmol/L) | 3.93±0.93 | 4.01±0.82 | 3.56±1.04^*#^ | <0.0001 |
| Triglyceride (mmol/L) | 1.01±0.49 | 1.03±0.81 | 0.85±0.45^*#^ | <0.0001 |
| HDL-c (mmol/L) | 1.00±0.25 | 1.03±0.26 | 0.97±0.36^#^ | 0.009 |
| LDL-c (mmol/L) | 2.31±0.0.77 | 2.42±0.69 | 2.02±0.83^*#^ | <0.0001 |
| Apo A (g/L) | 0.83±0.58 | 0.84±0.60 | 0.80±0.52 | 0.45 |
| Apo B (g/L) | 0.53±0.6 | 0.53±0.39 | 0.48±0.33^#^ | 0.02 |

Abbreviations: HDL-c, High density lipoprotein cholesterol; LDL-c, Low density lipoprotein cholesterol; Apo A, Apolipoprotein A; Apo B, Apolipoprotein B.

^*^ p value <0.05 vs non-cirrhosis; ^#^ p value <0.05 vs compensated cirrhosis.
